# Creep-Fatigue Failure Diagnosis

**DOI:** 10.3390/ma8115418

**Published:** 2015-11-16

**Authors:** Stuart Holdsworth

**Affiliations:** EMPA: Swiss Federal Laboratories for Materials Science and Technology Überlandstrasse 129, Dübendorf CH-8600, Switzerland; stuart.holdsworth@empa.ch; Tel.: +41-58-765-47-32

**Keywords:** failure diagnosis, creep-fatigue, material condition, mechanical analysis

## Abstract

Failure diagnosis invariably involves consideration of both associated material condition and the results of a mechanical analysis of prior operating history. This Review focuses on these aspects with particular reference to creep-fatigue failure diagnosis. Creep-fatigue cracking can be due to a spectrum of loading conditions ranging from pure cyclic to mainly steady loading with infrequent off-load transients. These require a range of mechanical analysis approaches, a number of which are reviewed. The microstructural information revealing material condition can vary with alloy class. In practice, the detail of the consequent cracking mechanism(s) can be camouflaged by oxidation at high temperatures, although the presence of oxide on fracture surfaces can be used to date events leading to failure. Routine laboratory specimen post-test examination is strongly recommended to characterise the detail of deformation and damage accumulation under known and well-controlled loading conditions to improve the effectiveness and efficiency of failure diagnosis.

## 1. Introduction

The diagnosis of failures invariably involves consideration of both the associated material condition and the results of a mechanical analysis of prior operating history. Material condition refers not only to a knowledge of the chemical composition and mechanical properties relative to those originally specified for the failed component, but also the appearance and extent of microstructural and physical damage responsible for failure. At the very least, the latter directs the investigator to the mechanism(s) of failure and to the type of mechanical analysis that should be adopted to corroborate the diagnosis. Typically, the required details relating to material condition and prior operating history are incomplete, and it is necessary to exploit the available evidence from both sources of information. Creep-fatigue failures in high temperature power plant components are good examples of this since the failure mechanism can be camouflaged by extensive oxidation and the detail of thermo-mechanical transients can be complex and are not always comprehensively gathered during operation.

Oxidation can disguise crack path details in such a way that the actual damage mechanism is no longer apparent, and valuable evidence can be lost by its removal. Furthermore, creep-fatigue damage development can be very material condition-dependent, being influenced not only by creep ductility, but also by creep strength and the way in which it has been attained; that is, by precipitation strengthening or by solid solution strengthening. For these reasons, the accurate microstructural characterisation of creep-fatigue damage often requires a knowledge of operating conditions (and the results of mechanical analysis) and the response of the material to thermo-mechanical fatigue loading (e.g., from laboratory testing experience). In this respect, the routine practice of laboratory specimen post-test examination is strongly advocated.

The following review concerns the diagnosis of creep-fatigue failures with due consideration to material condition and mechanical analysis of prior operating history.

## 2. Material Condition

### 2.1. Mechanism of Creep-Fatigue Cracking

**Figure 1:**
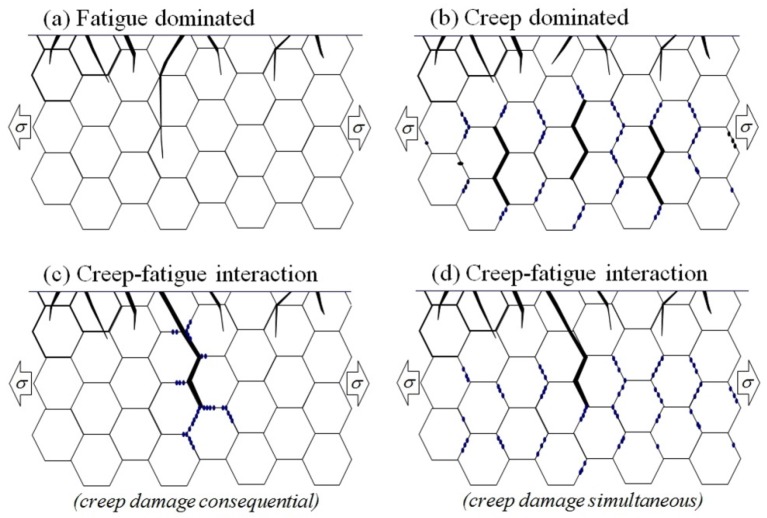
Creep-fatigue cracking mechanisms: (**a**) fatigue dominated; (**b**) creep dominated; (**c**) creep-fatigue interaction (due to “consequential” creep damage accumulation); (**d**) creep-fatigue interaction (due to “simultaneous” creep damage accumulation).

**Figure 2:**
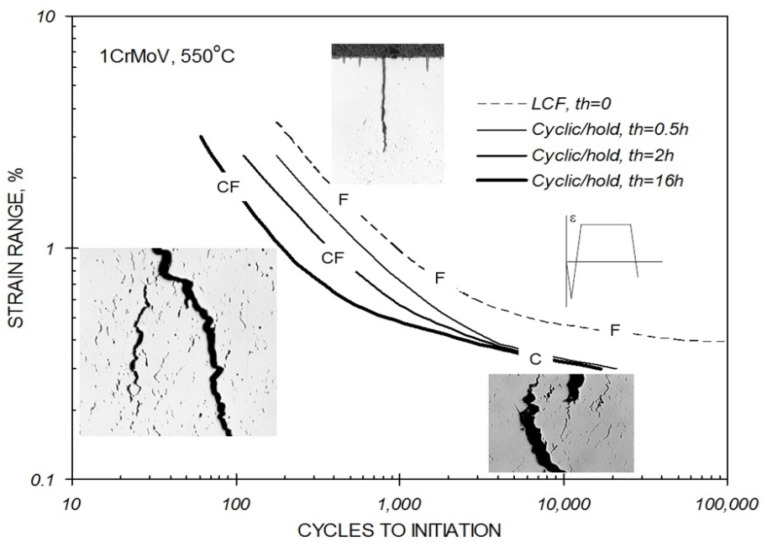
Influence of hold time on the cyclic/hold creep-fatigue endurance of 1CrMoV steel at 550 °C where crack development is identified as being pure creep (C), pure fatigue (F) or creep-fatigue (CF). LCF = Low Cycle Fatigue.

The development of creep-fatigue damage in most power plant steels depends on temperature, strain range, strain rate, hold time, and the creep strength and ductility of the material [1,2,3,4]. In the absence of a significant hold time (and/or at relatively high strain rates), crack initiation and growth is fatigue dominated, even at high application temperatures (Figure 1a). With increasing hold time (and/or decreasing strain rate) at high temperatures, the creep damage within the structure becomes increasingly influential, to the limit beyond which crack development becomes fully creep dominated (Figure 1b). At intermediate hold times and strain rates, fatigue cracking interacts with creep damage developing “consequentially” or “simultaneously” resulting in accelerated crack propagation and reduced crack initiation endurance (Figure 1c,d and Figure 2). The extent of any interaction increases with decreasing creep ductility [3]. The interaction of creep and fatigue is not limited to the accumulation of damage. Deformation interactions are also influential, and in a dominant way for a number of alloys [2].

### 2.2. Creep Ductility

**Figure 3:**
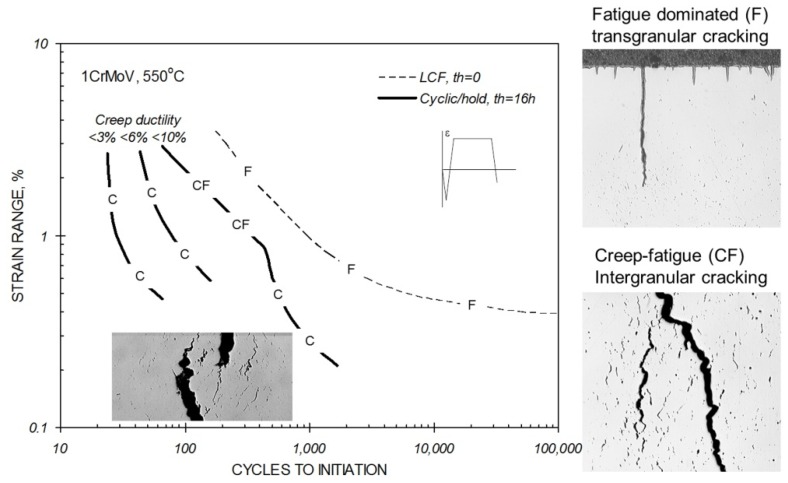
Influence of creep ductility on the cyclic/hold creep-fatigue endurance of 1CrMoV steel at 550 °C.

**Figure 4:**
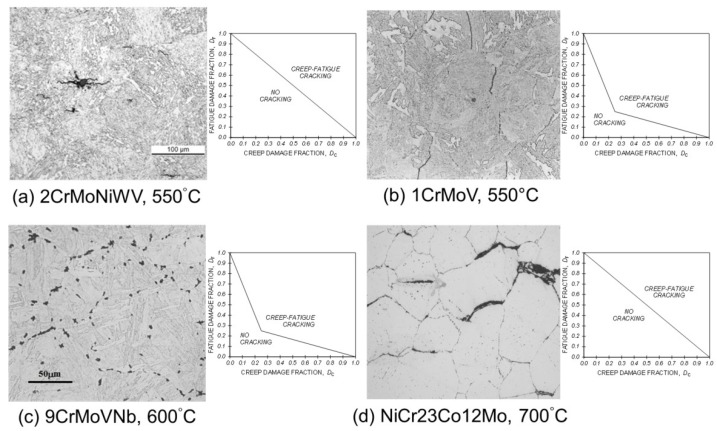
Creep damage development: (**a**) transgranular due to particle-matrix decohesion in creep ductile 2CrMoNiWV steel at 550 °C; (**b**) intergranular in a 1CrMoV steel at 565 °C; (**c**) intergranular in a 9CrMoVNb steel at 600 °C; (**d**) intergranular in a NiCr23Co12Mo alloy at 700 °C.

**Figure 5:**
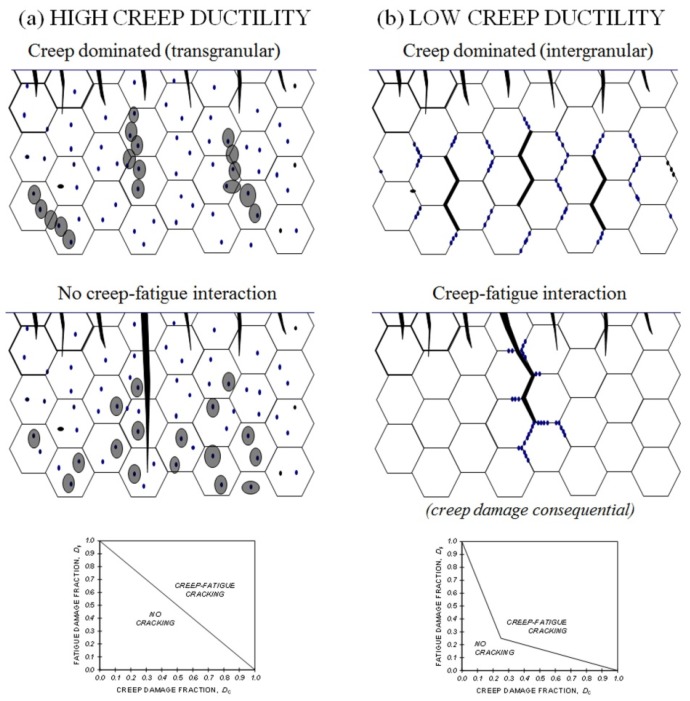
Influence of creep-ductility on creep-fatigue cracking mechanisms.

**Figure 6:**
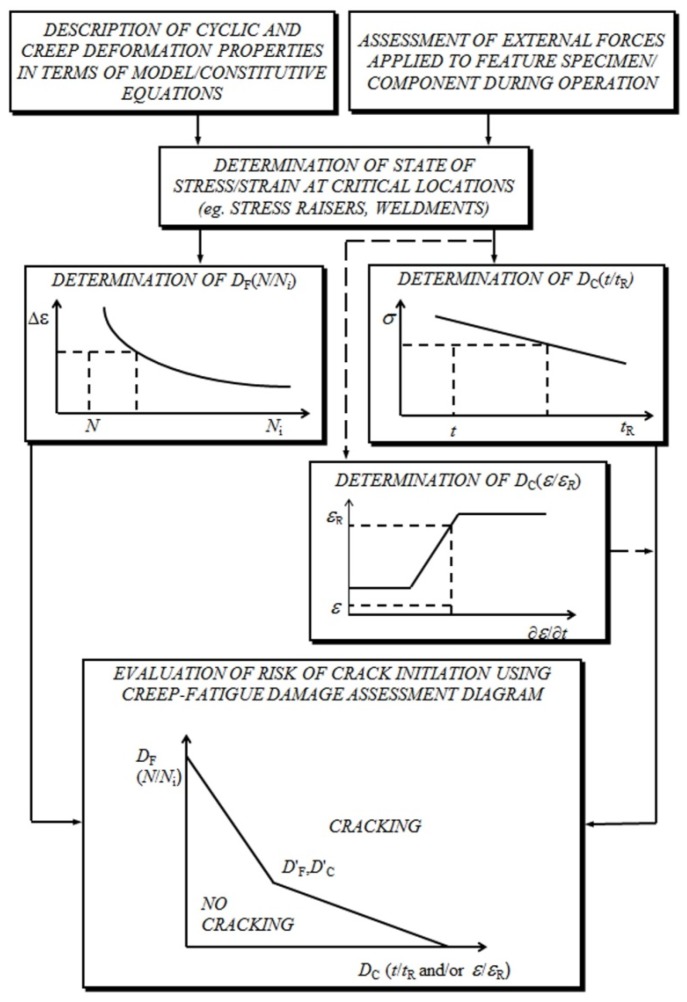
Generic flow diagram representing creep-fatigue crack initiation assessment procedure.

Creep ductility is influential in determining the extent of creep-fatigue interaction (Figure 3). When creep ductility is high, creep voids typically tend to form predominantly at inclusions as a consequence of particle matrix decohesion (Figure 4a), creep dominated cracking tends to be transgranular rather than intergranular (Figure 5a), and creep-fatigue failure is due to damage summation with insignificant interaction (linear damage summation, see inset with reference to Figure 6). When creep ductility is low, creep cavities typically form at grain boundaries, and the extent of creep-fatigue interaction can be high (Figure 5b).

In practice, the situation is not always as simple as this. Intergranular creep damage development is not restricted to low creep ductility (creep brittle) materials, that is, with creep-rupture ductility, *ε*_R_, of less than approximately five percent. While this is typical for precipitation-strengthened ferritic steels (Figure 4b,c), it can also occur in higher ductility solid solution strengthened alloys (e.g., Figure 4d). It is therefore important to be familiar with the pedigree and creep response of a material when making judgments concerning its creep-fatigue behaviour. 

### 2.3. Advanced Martensitic Steels

The development of creep damage in the more creep resistant of the advanced 9/11%Cr martensitic steels is not limited to the prior austenite grain boundaries and is not always as clear as is shown in Figure 4c. Often requiring very careful examination, very fine creep cavities can be observed to form also on lath and packet boundaries in this class of steel. This being the case, creep-fatigue cracking can appear to be transgranular, although crack development is actually along alternative creep-damaged boundaries [5].

Indeed, in this class of steel, the microstructural evidence appears to indicate that creep-fatigue deformation interactions are more important than creep-fatigue damage interactions [2,5,6,7]. Precipitation-strengthened steels such as the advanced 9/11%Cr martensitic steels typically cyclic soften during fatigue loading as a consequence of dynamic recovery and the development of a sub-grain structure. Sub-grains of a similar size develop during creep deformation, again as the consequence of dynamic recovery. The sizes of sub-grains that develop due to creep-fatigue loading are much greater [6,7], and provide a means of quantifying the extent of deformation interaction in steels that do not always exhibit classical evidence of creep-fatigue damage interaction [7]. This type of familiarity with material condition is invaluable for effective and efficient failure diagnosis.

## 3. Mechanical Analysis of Creep-Fatigue Cracking

### 3.1. Crack Initiation

While there are a number of published and in-house procedures available to assess the risk of creep-fatigue crack initiation in high-temperature components (e.g., [8,9,10,11]), most can be represented by the generic flow diagram shown in Figure 6. An important step in any creep-fatigue assessment procedure is a determination of the state of stress and strain at the critical location in the component. This requires a knowledge of the external forces and thermal transients experienced by the structure during service operation, and representations of the cyclic and creep deformation properties of the material(s) of construction in terms of model constitutive equations (e.g., [12]). Irrespective of whether the local stress-strain state is determined by approximate analytical solutions or finite element analysis (FEA), the constitutive model options are generally the same.

With this information, fatigue and creep damage fractions can be determined. Fatigue damage fraction is commonly determined in terms of cycle number fraction, *N*/*N*_i_(Δ*ε*), where *N*/*N*_i_(Δ*ε*) can be the low cycle fatigue (LCF) or the cyclic/hold creep-fatigue test crack initiation endurance (depending on procedure). The method of determination of creep damage fraction can depend on whether it is accumulated due to primary (directly applied) or secondary (self-equilibrating) loading. Regardless of the approach adopted, the material properties required are derived from the results of conventional creep-rupture tests.

With a knowledge of the fatigue and creep damage fractions accumulated per cycle at the component critical location, it is then possible to determine the creep-fatigue crack initiation endurance; for example, by means of a damage summation construction (Figure 6, also [13]). Typically such diagrams are constructed from the results of cyclic/hold (LCF with hold time) creep-fatigue tests or thermo-mechanical fatigue (TMF) tests, which indicate the extent of any creep-fatigue interaction of the material of interest under precisely known thermo-mechanical boundary conditions (e.g., insets in Figure 4 and Figure 5).

### 3.2. Crack Development

**Figure 7:**
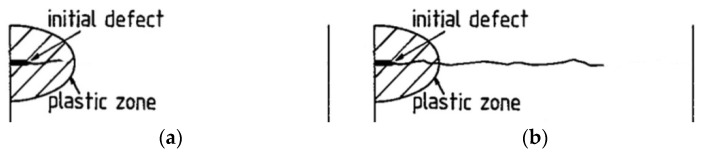
Schematic representation of (**a**) short-crack growth within a cyclic plastic zone; and (**b**) long crack growth beyond the boundary of a cyclic plastic zone.

Crack development due to creep-fatigue loading may occur (i) within the confines of a cyclic plastic zone (when the crack is physically small, *i.e.*, <~5 mm, more typically <~2 mm) or (ii) beyond the limits of the size of the cyclic plastic zone, *r*_p_ (when the crack is long and loading is nominally elastic), Figure 7.

In the long-crack regime (Figure 7b), creep-fatigue crack growth is typically represented by fatigue and creep crack growth rate characteristics [11,14], *i.e.*,

(1) $${\left(da/dN\right)}_{\text{CF}}=\;{\left(da/dN\right)}_{\text{F}}+\;{\left(da/dN\right)}_{\text{C}}$$

where

(2) $${\left(da/dN\right)}_{\text{F}}=A\left(T,v,t_{\text{h}}\right)\cdot {\left(\Delta K_{\text{eq}}\right)}^{\text{m}}$$

and

(3) $${\left(da/dN\right)}_{\text{C}}=\;\int_{0}^{t_{\text{h}}}D\left(\varepsilon_{R}\right)\cdot {\left(C^{\text{*}}\right)}^{\gamma }\cdot dt/v$$

**Figure 8:**
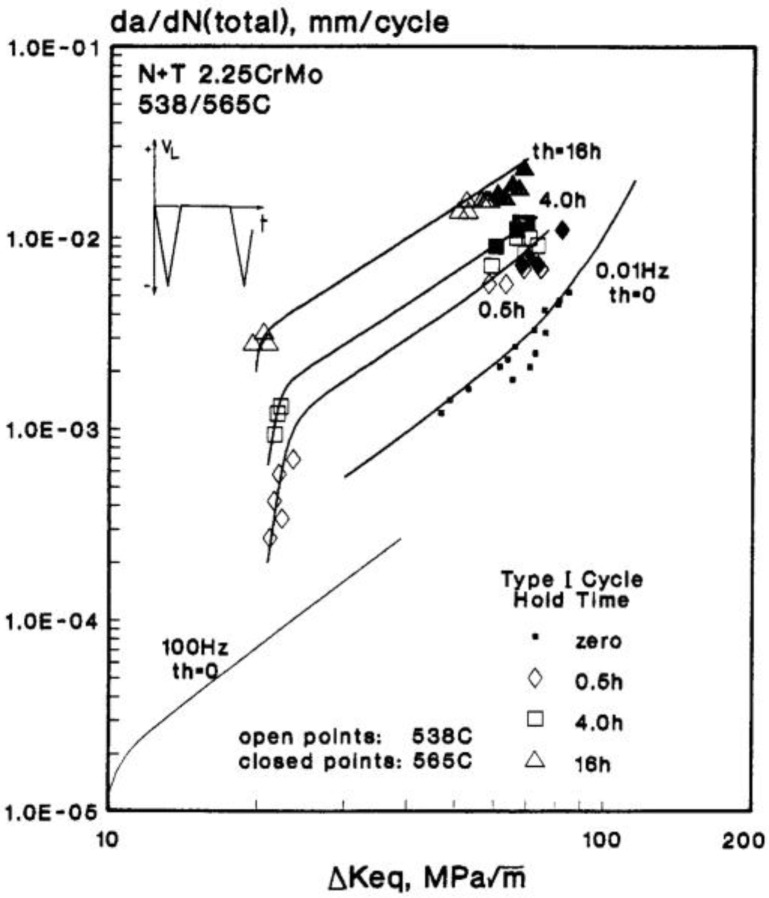
Long crack cyclic/hold creep-fatigue crack growth test data for 2¼CrMo cast turbine steel at 538/565 °C. N + T = Normalised and Tempered.

Creep-fatigue-oxidation interaction is accommodated through the *A*(*T*, *v*, *t*_h_) function in Equation (2) which accounts for any influence of prior creep and oxidation damage at the crack tip, and may be determined experimentally (e.g., Figure 8) [14]. For steady-state creep conditions ahead of the crack tip, the creep rate dependent C* parameter provides an acceptable size and geometry-independent function for correlating creep crack growth rates for long cracks [15]. 

In the short crack regime (Figure 7a), creep-fatigue crack growth rates may be expressed as a function of total strain range, as in Equation (4) [16,17,18,19], although other correlating parameters may be employed [19].

(4) $$da/dN=\;{\text{B}}^{\prime }\cdot {\left(\text{Δ}\varepsilon \right)}^{\text{b}}\cdot a^{Q}\cdot {\left(1-D_{\text{C}}\right)}^{-2}\;$$

**Figure 9:**
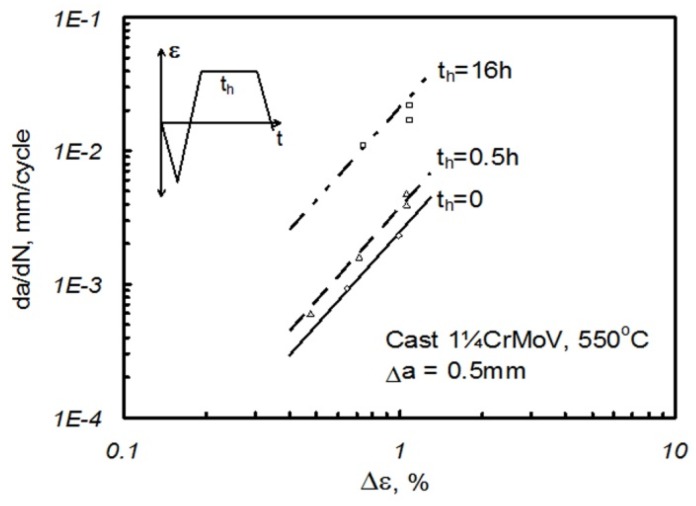
Comparison of short crack growth rates after 0.5 mm crack extension from notch root in large single edge notched bend (SENB) feature specimen creep-fatigue tests on cast 1¼CrMoV steel at 550 °C.

Typically in Equation (4), *Q* = 1. For advanced martensitic steels, it has been shown that replacing *D*_C_ by a microstructural condition parameter (*Φ*) can be more appropriate [7]. In this case, *Φ* is a function of the sub-grain size, and reflects the deformation state due to creep-fatigue loading (see Section 2.3). An example of short-crack creep-fatigue crack growth data for a cast 1¼CrMoV steel at 550 °C is shown in Figure 9. Data for martensitic steels are given in [7,19].

## 4. Post-Test Examination

An effective quantitative interpretation of the evidence associated with a component failure requires familiarity with the microstructural and damage conditions of the constituent material(s) under known and well-controlled loading conditions. For this reason alone, it is important that laboratory mechanical test specimens are routinely subjected to systematic post-test examination.

A classic example is the classification of fatigue fracture appearance in terms of *K*_mean_, Δ*K,* and temperature. The collection of this knowledge as an integral part of a fatigue crack growth testing campaign enables rapid indications of loading conditions responsible for service failures (e.g., [20]), in particular those at temperatures where crack surface oxidation is not a complicating issue. This attention to detail can reveal the appearance of beachmarks associated with different types of loading transient which can be invaluable information when beachmark analysis is being used to track the history of sub-critical crack propagation during service to identify the incident responsible for the initiation of cracking.

Needless to say, the concept of post-test examination is not always so revealing at higher temperatures. The coverage of important features with the products of oxidation can disguise the explanation for failure, although oxide thickness measurements provide a useful indication of time of exposure at temperature [21,22,23], and thereby the basis for oxide dating.

### 4.1. Oxide Dating

**Figure 10:**
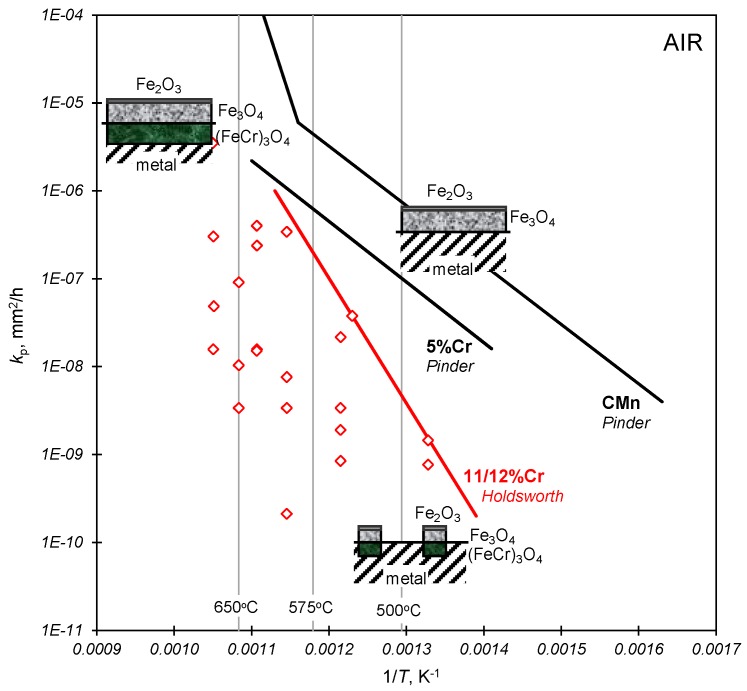
The influence of chromium content on oxidation kinetics for a range of power plant steels in air [23] (Insets indicate oxidation mechanisms for the low- and high-alloy steels)*.*

Measurements from the post-test examination of specimens tested under known conditions provide invaluable evidence for subsequent failure diagnosis. One example of this is the collection of oxidation data which can then be used to form the basis of an oxide dating protocol and to determine growth law constants, e.g.,

(5) $$x^{2}=\;k_{\text{p}}\cdot t$$

where *k*_p_ is a function of material, temperature and surface condition. While the influence of surface conditions such as roughness and the states of activity and stress/strain are significant, use of upper-bound relationships of the type shown in Figure 10 are invaluable for dating purposes, providing there is evidence (in the form of an outer haematite layer) that the oxide, whose thickness is to be measured, is intact.

The results of laboratory specimen post-test examination are most effective for failure diagnosis when quantified, and the following sections review the way in which this can be achieved for creep, fatigue, and creep-fatigue damage.

### 4.2. Creep Damage Assessment

Creep damage condition can be represented in terms of feature type, development state, or size.

#### 4.2.1. Type

Damage may be simply characterised as either (i) cavities on grain/block/lath boundaries [24,25,26] or (ii) voids arising from particle/matrix decohesion. When material creep ductility is low (Figure 5b), boundary cavities occur at a relatively early stage, thereby providing a means of assessing remaining life. Creep voids forming due to particle/matrix decohesion generally do so late in life (Figure 5a), typically in creep ductile materials when the ductility is almost exhausted. The particles are typically inclusions and carbides located at intra-granular sites.

#### 4.2.2. Development

Creep damage is most commonly characterized in terms of a quantity representing its extent or degree of development, e.g., by a classification based on reference micrographs [24], or a simple density measurement of cavities/mm^2^ [25,26,27]. The latter has the advantage that it can be used to quantify the density of one or both types of damage defined above, in particular in the vicinity of non-uniform stress fields; for example, adjacent to stress concentrations or cracking [28]. For boundary cavitation, cavity number is alternatively quantified by continuous cavity line length [25], or A-parameter [26].

#### 4.2.3. Size

The characterisation of damage size may involve sizing of (i) the damage feature (e.g., cavity diameter); or (ii) the damage zone. In reality, the measurement of cavity/void size [27] is not widely practiced, primarily because of the high sensitivity of damage feature diameter and its dependent parameters on metallographic surface preparation. A knowledge of microcrack length is required to assign advanced damage categories [25]. Damage zones may be sized in terms of parameters of the type developed in [28].

### 4.3. Fatigue Damage Assessment

**Figure 11:**
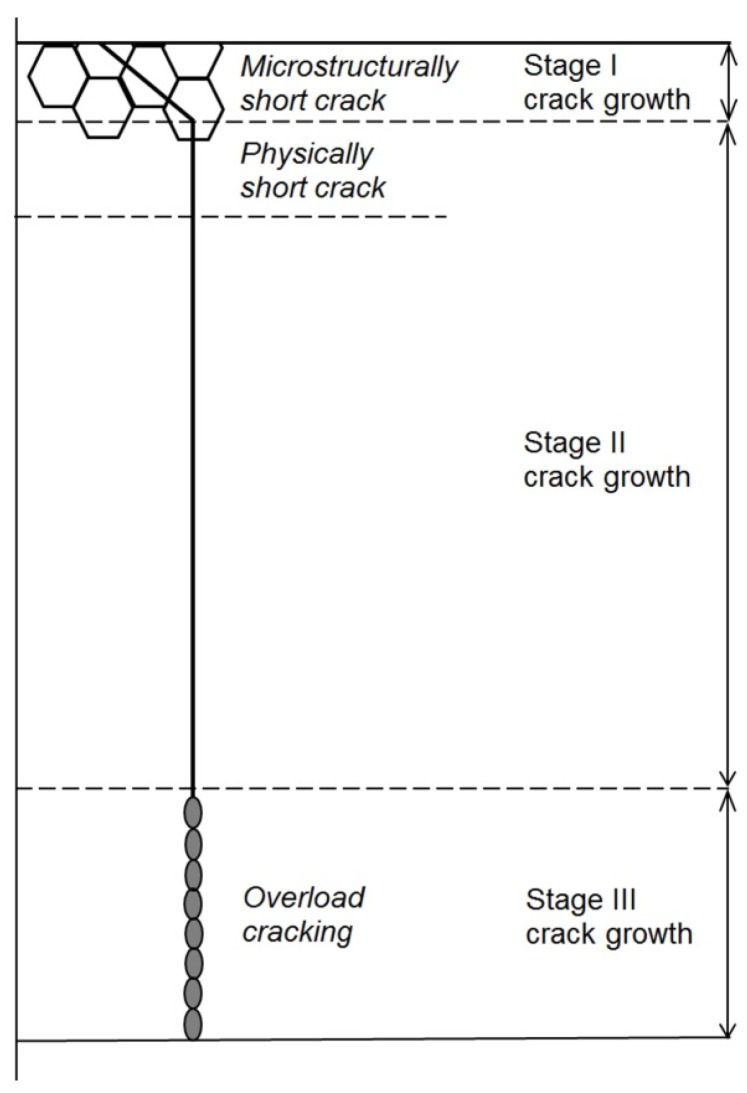
Schematic representation of fatigue crack development.

Microstructurally short Stage I fatigue cracks typically extend along persistent slip bands [29] to a depth of 1–2 grain diameters before becoming Stage II cracks propagating in a transgranular manner normal to the maximum principal stress (Figure 11) [30,31]. 

In Figure 11, reference is also made to physically short cracks, the maximum length of which may be defined by a Kitagawa diagram construction [32], e.g., ~250 µm for 1CrMoV rotor steel.

Typically on the surface of a part subjected to cyclic loading, many microstructurally short fatigue cracks are formed, a relatively small proportion of which will develop to become physically short cracks, depending on strain amplitude. Also depending on strain amplitude, one or more physically short fatigue crack will become dominant, extending to become a long crack responsible for failure [33]. This sort of damage distribution can be quantified (e.g., as crack densities [34,35]).

### 4.4. Creep-Fatigue Damage Assessment

The assessment of creep-fatigue damage has tended to focus on materials for which creep damage is intergranular, and for circumstances represented by Figure 1b–d (e.g., [34,35,36,37]). In these circumstances, intergranular damage may be quantified as a function of *L*_f_ [36] or *L*_f_/*L*_t_ [34]. In both cases, measurements are made away from the main crack in order to estimate the homogeneous damage due to creep, without interaction with major cracks. Creep-fatigue damage may then be quantified as a function of cracked grain boundary fraction and surface crack density [34].

The traditional view of creep-fatigue damage development and interaction involves the development of transgranular fatigue cracking from the surface and intergranular creep damage from sub-surface to the point where the intensity of grain boundary damage is sufficient to deflect crack propagation onto the grain boundaries (e.g., Figure 1d) [35,38]. It is now appreciated that this type of creep-fatigue damage interaction does not always occur for creep ductile alloys (e.g., Figure 5), and that creep-fatigue response for the newer advanced 9/11%Cr steels can be strongly influenced by both deformation and damage interactions [2,6,7].

**Figure 12:**
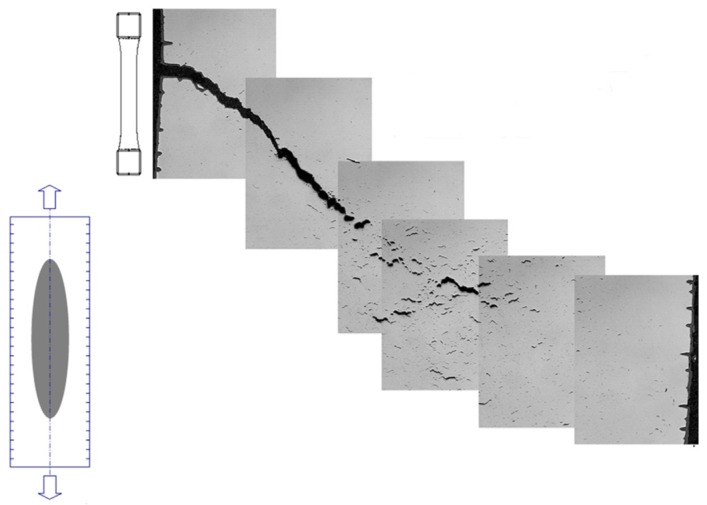
Creep-fatigue crack development in a 1CrMoV rotor steel at 565 °C.

A good example of the development of creep-fatigue damage in a laboratory specimen of 1CrMoV at 565 °C is shown in Figure 12. Multiple microstructurally short fatigue crack development occurs at the surface, while creep damage evolves from the center of the specimen. When the dominant fatigue crack meets the creep damage zone advancing from the axis, interaction occurs in the way represented in Figure 1d.

**Figure 13:**
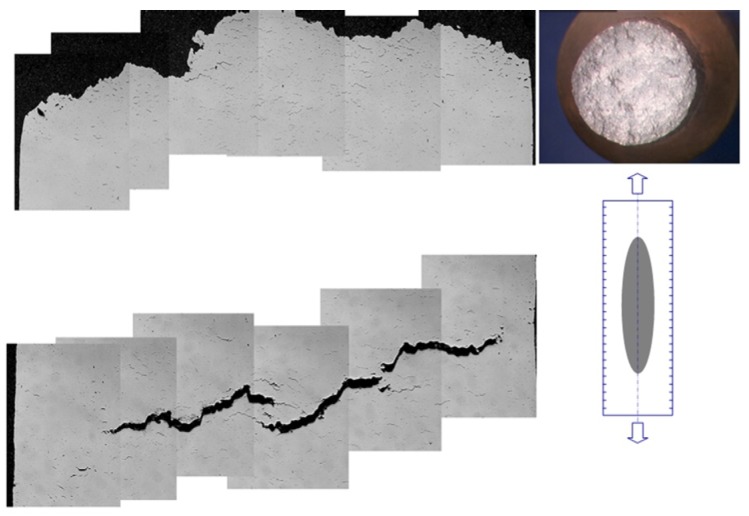
Creep dominated crack development in a 1CrMoV rotor steel (the upper and lower profiles are from two specimens following thermo-mechanical fatigue testing under identical conditions: the upper one breaking open when cold (due to the high density of internal creep cavities); and the lower one remaining unbroken).

It is important not to underestimate the influence of oxidation on creep-fatigue crack development. While oxidation can enhance the rate of cracking, it can also consume any microcrack development to the point where thermo-mechanical crack propagation is entirely creep dominated (e.g., Figure 13). In such cases, strain-enhanced oxidation can consume all evidence of microcrack development and become detached by spalling due to large cyclic thermal transients.

It has already been acknowledged that microstructural evidence of a strong creep-fatigue interaction is not always only apparent in the form of physical damage (*i.e.*, as cavities and cracking). For example, the strong deformation interaction exhibited by some advanced martensitic 9/11%Cr steels can be more readily revealed by examination of the sub-grain microstructure [6,7]. Familiarity with the metallurgical characteristics of the material of the failed component is therefore invaluable for effective and efficient failure diagnosis.

## 5. Concluding Remarks

Failure diagnosis invariably involves consideration of both the associated material condition and the results of a mechanical analysis of prior operating history. This review has focused on these aspects with particular reference to creep-fatigue failure diagnosis.

Creep-fatigue cracking can be due to a spectrum of loading conditions ranging from pure cyclic to mainly steady loading with infrequent off-load transients. These require a range of mechanical analysis approaches, a number of which are reviewed. The effectiveness and efficiency of the diagnosis of failures due to creep-fatigue loading are enhanced by a familiarity with the characteristics of the material of the failed component which can come from the routine post-test examination of laboratory specimens.

The concept of routine laboratory specimen post-test examination to quantitatively characterise the detail of deformation and damage accumulation under known and well-controlled loading conditions is strongly advocated, and illustrated with examples.

## Nomenclature

*a*	Crack length
*A*(*T*,ν,*t*_h_)	Constant in mid-Δ*K* fatigue crack growth rate equation
*b*	Strain range exponent in short-crack creep-fatigue crack growth equation
*B'*	Constant in short-crack creep-fatigue crack growth equation
C, F, CF	Creep; Fatigue; Creep-fatigue
*C**	Parameter characterising stress and strain rate fields at tip of crack in material deforming due to creep
d*a*/d*N*	Total crack growth rate (per cycle)
(d*a/*d*N*)_C_, (d*a*/d*N*)_F_	Crack growth rate (per cycle) due to creep and fatigue respectively
*D*_C_, *D*_F_	Total creep damage fraction; Total fatigue damage fraction
*D*(*ε*_R_)	Constant in creep crack growth law, dependent on creep-rupture ductility
*k*_p_	Parabolic oxide growth law constant
*K*, Δ*K*	Stress intensity factor; Range of stress intensity factor
*K*_eq_	Equivalent stress intensity factor (*K* with a plasticity correction, similar to *J*)
LCF	Low cycle fatigue
*L*_f_, *L*_t_	Cumulative length of cracked grain boundaries per unit length of section; Total length of grain boundaries in the same area (being characteristic of the grain size and shape)
*J*	Parameter characterising stress and strain fields at tip of crack in material deforming plastically
*m*	Exponent in mid-Δ*K* fatigue crack growth rate equation
*N*, *N*_i_	Number of cycles; Number of cycles to crack initiation
N + T	Normalised and tempered
*Q*	Crack size exponent in short-crack creep-fatigue crack growth rate equation
*r*_p_	Size of cyclic plastic zone
SENB	Single edge notched bend (specimen)
*t*, *t*_h_, *t*_R_	Time; Hold time; Time to creep-rupture
TMF	Thermo-mechanical fatigue
*x*	Oxide thickness
*ε*, Δ*ε*	Strain; Strain range
*ε*_R_	Creep-rupture ductility
*Φ*	Microstructural condition parameter
*γ*	*C** exponent in creep crack growth law
*ν*	Frequency
